# The effect of the cold pressor test on a visually evoked cerebral blood flow velocity response in patients with migraine

**DOI:** 10.1186/1129-2377-14-S1-P120

**Published:** 2013-02-21

**Authors:** A Fabjan, J Vidmar, B Musizza, FF Bajrović, M Zaletel, M Štrucl

**Affiliations:** 1 Institute of Physiology, Medical Faculty, University of Ljubljana, Slovenia; 2 Jožef Stefan Institute, Ljubljana, Slovenia; 3 Institute of Pathophysiology, Medical Faculty, University of Ljubljana, Slovenia; 4 Department of Neurology, University Medical Centre of Ljubljana, Slovenia

## Introduction

Our previous study has shown that visually evoked cerebral blood flow velocity response (VEFR) is increased during cold pressor test (CPT) in healthy human subjects [1]. Our results supported the assumption that the activity of neurovascular coupling (NVC) increases during tonic pain stimulus due to the modulatory influence of activated subcortical structures [1].

## Purpose

In the present study, we investigated the hypothesis that the effect of tonic pain stimulus on NVC is altered in patients with migraine.

## Methods

23 healthy subjects (10 males, mean age 36 ± 10 y; 13 females, mean age 38 ± 15 y) and 29 patients with migraine (8 males, mean age 39 ± 13 y; 21 females, mean age 36 ± 11 y) participated in the study. Arterial blood pressure, heart rate, end-tidal carbon dioxide partial pressure and blood flow velocities in the right posterior and the left middle cerebral artery were continuously measured. VEFR was calculated as relative increase in blood flow velocity in the posterior cerebral artery from average values during last 5 seconds of stimulus OFF period to average values during last 10 seconds of following stimulus ON period. Three consecutive experimental phases were compared: basal, CPT and recovery.

## Results

In healthy subjects, during CPT, end-diastolic VEFR increased from 20.2 to 23.6% (p<0.05) and subsequently decreased to 17.7% in recovery phase (p<0.05). In patients with migraine, no statistically significant change in end-diastolic VEFR was observed between phases (p>0.05). Additionaly, the differences in end-diastolic VEFR between the basal phase and the CPT phase and between the CPT phase and the recovery phase were statistically significantly higher in healthy subjects than in patients with migraine (p<0.05).

## Conclusion

Our results are consistent with the assumption that there is a lack of effect of tonic pain on the activity of NVC due to dysfunction of modulatory subcortical pain structures in patients with migraine.
